# Involvement of calcium channels in the regulation of adipogenesis

**DOI:** 10.1080/21623945.2020.1738792

**Published:** 2020-03-16

**Authors:** Mingzhu Zhai, Dazhi Yang, Weihong Yi, Wuping Sun

**Affiliations:** a Huazhong University of Science and Technology Union Shenzhen Hospital and the 6th Affiliated Hospital of Shenzhen University Health Science Center, Shenzhen, China; b Department of Orthopaedics, Huazhong University of Science and Technology Union Shenzhen Hospital, Shenzhen, China; c Department of Pain Medicine and Shenzhen Municipal Key Laboratory for Pain Medicine, Huazhong University of Science and Technology Union Shenzhen Hospital, Shenzhen, China

**Keywords:** Calcium channels, calcium signalling, trp channels, adipogenesis, adipocyte differentiation, proliferation, obesity, body weight

## Abstract

As an important second messenger in adipocytes, calcium ions (Ca^2+^) are essential in regulating various intracellular signalling pathways that control critical cellular functions. Calcium channels show selective permeability to Ca^2+^ and facilitate Ca^2+^ entry into the cytoplasm, which are normally located in the plasmatic and intracellular membranes. The increase of cytosolic Ca^2+^ modulates a variety of signalling pathways and results in the transcription of target genes that contribute to adipogenesis, a key cellular event includes proliferation and differentiation of adipocyte. In the past decades, the involvement of some Ca^2+^-permeable ion channels, such as Ca^2+^ release-activated Ca^2+^ channels, transient receptor potential channels, voltage-gated calcium channels and others, in adipogenesis has been extensively explored. In the present review, we provided a summary of the expression and contributions of these Ca^2+^-permeable channels in mediating Ca^2+^ influxes that drive adipogenesis. Moreover, we discussed their potentials as future therapeutic targets.

## Adipogenesis & obesity

**Figure 1.**
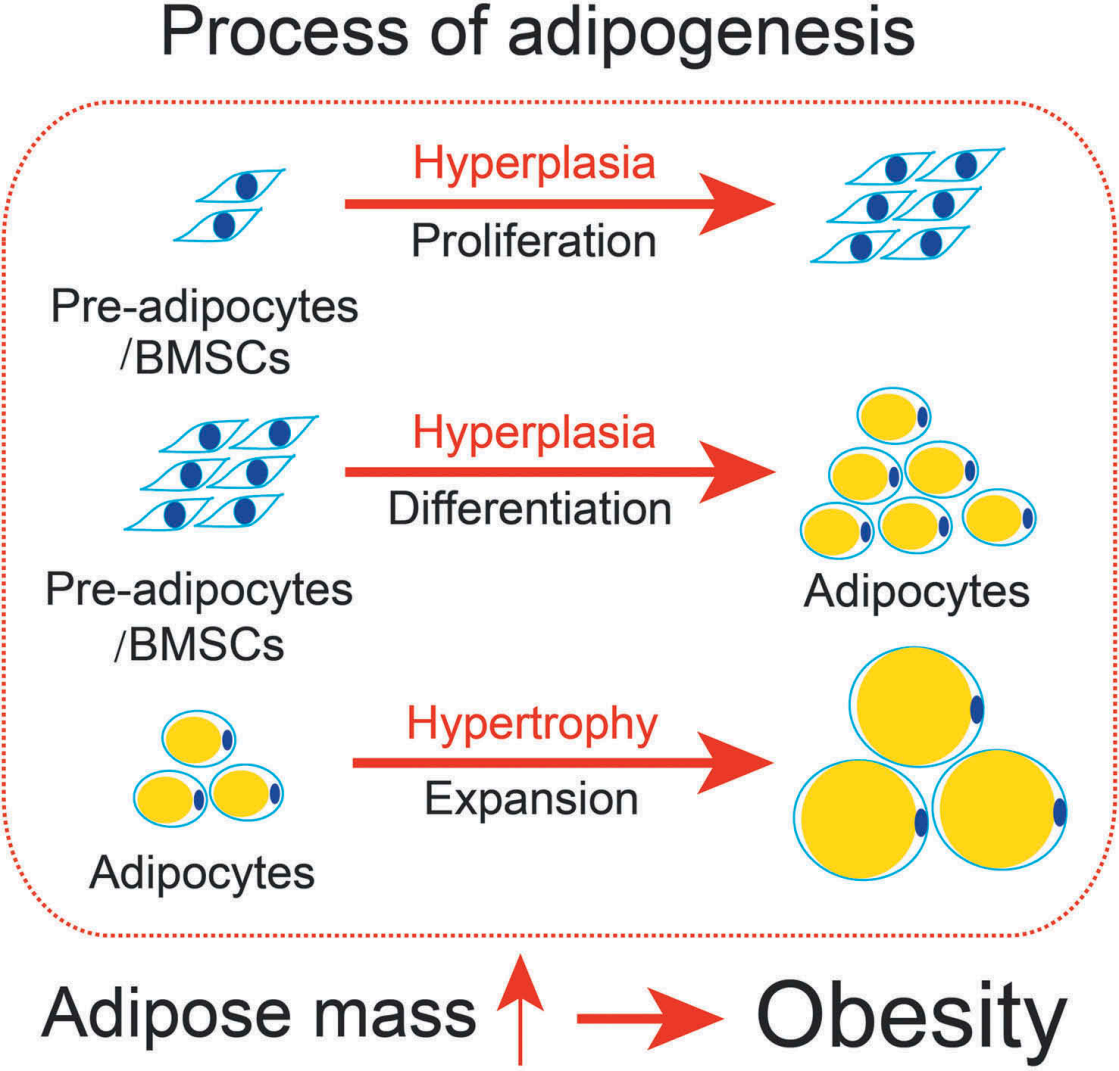
The schematic diagram of the process of adipogenesis

Obesity is believed to be the result from an imbalance between energy intake and energy expenditure [1], and it is characterized by increased adipose tissue mass (fat deposition) that results from increased fat cell size (hypertrophy) and number (hyperplasia), suggesting that the main contributor to obesity is adipose tissue (Figure 1) [2]. Adipose tissue has been reported plays a fundamental role in the maintenance of energy homoeostasis and diverse biological processes, such as haematopoiesis, insulin sensitivity, vascular remodelling, immune response and metabolic derangements associated with obesity [3]. Two types of adipose tissue have been reported existing in humans and mammals, which are white adipose tissue (WAT) and brown adipose tissue (BAT), and function in the opposite way to maintain energy homoeostasis. WAT stores excess energy as triglyceride in lipid droplets, while BAT provides basal and inducible energy consumption by utilizes stored lipid droplets to generate heat in a process that is known as thermogenesis [4,5].

Adipocytes are derived from pre-adipocytes/bone marrow mesenchymal stem cells (BMSCs). The processes of adipogenesis, including two steps, hyperplasia (proliferation and differentiation, increased fat cell number) and hypertrophy (expansion, increased fat cell size). Upon adipogenesis, the increased adipose tissue mass (fat deposition) causes obesity.

Adipocytes are derived from mesenchymal precursor cells [6]. The processes of adipogenesis involve a variety of cellular processes (Figure 1), including division, proliferation, expansion, and differentiation from pre-adipocytes to mature adipocytes [7]. It has been reported that remarkable changes occur during adipocyte differentiation in both morphology and molecular levels. For instance, the fibroblast-like preadipocytes is morphologically rounded up and the elevated expression of mRNAs including lipoprotein lipase and several transcriptional factors, such as CCAAT-enhancer-binding protein β (C/EBPβ) and C/EBPδ, followed by the increased expression of C/EBPα and peroxisome proliferator-activated receptor γ (PPARγ), which upregulate the expression of genes that characterize the adipocyte phenotype [8]. During the process of adipogenesis, lipid-droplets start to be generated and appear in the cytoplasm, and over time, these droplets fuse into one or several large droplets. Adipogenesis has also been reported to be involved in various physiological functions. White adipose tissue (WAT) has been previously reported that plays important roles in insulation, mechanical support and as a major tissue type for energy storage [9]. Mature adipocytes have also been demonstrated to show hormonal and sympathetic functions [10]. BAT has been reported in adult humans to function as the main type of thermogenic tissue in response to generate heat to provide basal and inducible energy consumption [11,12]. Under positive energy balance, the excessive energy from food intake leads to the accumulation of triglycerides in WAT by lipogenic enzymes. By contrast, when under negative energy balance, lipases hydrolyse the excessive lipid drops into free fatty acids and/or glycerol which reserve as free energy circulating in the blood to muscle, liver, BAT and other tissues [13]. Excessive accumulation of triglycerides will induce obesity which affects normal adipocyte function and elevates the risk in the development of other metabolic disorders [14]. Therefore, investigating the underlying mechanisms in regulating adipogenesis is essential to understand and prevent obesity and related metabolic disorders.

## Calcium signalling in adipogenesis

Calcium signalling plays a vital role in maintaining normal cellular functions, including proliferation, differentiation, homoeostasis, and apoptosis. The level of total body calcium is normally regulated. About 98% of total body calcium is stored in the skeleton as calcium phosphate. The calcium concentration of the extracellular fluid (ECF) is about 2.2–2.6 mmol/L in the form of total calcium, and about 1.3–1.5 mmol/L in the form of free calcium ions (Ca^2+^) [15]. The calcium concentration of intracellular fluid (ICF) is about 50–200 nmol/L, depending on the cell types, which is 20,000 to 100,000 times less than that in the ECF [16]. Therefore, the intracellular Ca^2+^ functions as an excellent messenger molecule that is widely involved in various cellular events, including generating action potentials, regulating enzyme activity, and bridging the extracellular and intracellular signal transduction [17].

Calcium channels play fundamental roles in regulating the concentration of cytosolic Ca^2+^, which could trigger a sudden increase of the cytosolic Ca^2+^ level up to 500–1000 nm/L. The cytosolic Ca^2+^ rises either by entering the cell through Ca^2+^-permeable ion channels on the plasma membrane, such as voltage-gated Ca^2+^ channels and transient receptor potential (TRP) channels and/or by release from the intracellular Ca^2+^ stores, such as the ER and mitochondria [18]. Once the action potentials generated or the signal transduction is completed, the cytosolic Ca^2+^ is then quickly removed from cytoplasm by various types of Ca^2+^ pumps, including the Ca^2+^-ATPase and Na^+^/Ca^2+^ exchanger on the plasma membrane pumping Ca^2+^ out of cells, and Sarco/endoplasmic reticulum Ca^2+^ pump (SERCA) on the ER membrane and the mitochondria calcium uniporter (MCU) on the mitochondria membrane, which pump Ca^2+^ back into the ER and mitochondria, respectively [19,20]. The timing and increasing level of cytosolic Ca^2+^ is precisely regulated by these proteins to balance the intracellular Ca^2+^ homoeostasis, which is fundamental for maintaining normal cell functions [17].

Calcium signalling is also a vital event in the process of adipogenesis, as it regulates various fundamental processes including proliferation, differentiation, energy metabolism and obesity. Cytosolic Ca^2+^ has been implicated in regulating adipocyte differentiation [21], which plays a key role in metabolic derangements associated with obesity in humans. Evidence have revealed that cytosolic Ca^2+^ concentrations are increased in 3T3-L1 pre-adipocytes after Ca^2+^-ATPase inhibitor treatment, such as thapsigargin, which in turn efficiently inhibits adipocyte differentiation, and impairs specific gene expression of adipocytes and reduces the accumulation of lipid drops [22,23]. These inhibitory effects could be also induced by either enhancing the activity of calcineurin, a Ca^2+^-dependent phosphatase [24] or by activation of calcineurin effectors, such as NFAT [25]. By contrast, cyclosporine A (CsA) treatment has been proved that inhibits calcineurin activity and increases adipocyte differentiation and lipid accumulation in 3T3-L1 pre-adipocytes [24]. These results are consistent with data that the treatment of CsA promotes obesity development in humans [26]. These results demonstrated that the elevation of cytosolic Ca^2+^ levels has negative effects on 3T3-L1 adipocyte differentiation similar to that seen in other cell types [27].

Extracellular Ca^2+^ is also involved in the modulation of adipogenesis. It has been reported that low extracellular Ca^2+^ promotes adipogenesis and high extracellular Ca^2+^ attenuates adipogenesis [28] in 3T3-L1 pre-adipocytes, which is consistent with the data observed in rats when feeding with dietary calcium [29,30]. Extracellular calcium modulates brown adipocyte differentiation as well. Low extracellular Ca^2+^ accelerates differentiation and high extracellular Ca^2+^ suppresses differentiation in mouse brown adipocytes [31]. Moreover, ionomycin-induced the increase of cytosolic Ca^2+^ enhances the proliferation of primary mouse bone marrow mesenchymal stem cells (BMSCs) but not differentiation [32]. And high extracellular Ca^2+^ promotes the proliferation of BMSCs via a calcium-sensing receptor and ERK signalling pathway [33]. However, several papers have reported that high extracellular Ca^2+^ enhances adipogenesis probably through L-type Ca_v_ channel [34] and calcium-sensing receptor [35] in porcine BMSCs. These contradictory data indicate the complicated mechanisms of calcium signalling in the regulation of adipogenesis. The mechanisms that govern the levels of intracellular Ca^2+^ involve membrane receptors, signalling molecules, and a diverse array of Ca^2+^ channels. In the next session, we summarized the calcium channels involved in regulating the concentration of cytosolic Ca^2+^ and adipogenesis.

## Ca^2+^ release-activated calcium channels in adipogenesis

Ca^2+^ influx via store-operated Ca^2+^ channels (SOCs) in the plasma membrane provides increased cytosolic Ca^2+^ level and sustains the activity of several intracellular enzymes including calcineurin, which is critical for adipogenesis [36]. SOCs channels are activated by a mechanism critically dependent on the depletion of endoplasmic reticulum (ER) Ca^2+^ stores [37], which is widespread in adipocyte tissue [38]. Selective calcium release-activated Ca^2+^ (CRAC) channels are one type of SOCs. A well-described mechanism of extracellular Ca^2+^ entering adipocytes is through the CRAC channel, which is found by stromal interaction molecule 1 (STIM1) [37] and Orai1 proteins [39,40]. It is known that the CRAC channel contains two components: Orai1, the pore-forming subunit, which is situated in the plasma membrane and regulates Ca^2+^ entry, and STIM1, the regulatory subunit located in the ER membrane. STIM1 function as a Ca^2+^ sensing protein monitoring the fluctuation of Ca^2+^ levels in ER, which normally spread out in the ER membrane and oligomerize during the low level of Ca^2+^. Orai1 and STIM1 function together to induce Ca^2+^ influx from the extracellular space into intracellular fluid, which is termed store-operated Ca^2+^ entry (SOCE) [41].

The functional presence of STIM1 and ORAI1 has also been confirmed in many non-excitable cell types [38]. It has been reported that the up-regulation of STIM1 increases Ca^2+^ influx via CRAC channels and inhibits the 3T3-L1 adipocyte differentiation [42]. These results are consistent with the data by over-expression of STIM1 in 3T3-L1 pre-adipocytes, which increases Ca^2+^ influx and inhibits 3T3-L1 adipocyte differentiation, without affecting their proliferation and growth arrest. The over-expression of STIM1 also induces the down-regulation of adiponectin and C/EBPα. Besides, the down-regulation of endogenous STIM1 promotes 3T3-L1 adipocyte differentiation, resulting in the up-regulation of C/EBPα and adiponectin [42]. Another study has demonstrated that STIM and SOCE also play an important role in the adiposity of *Drosophila* [43]. Impairment of STIM1, the core component of SOCE, causes adiposity in *Drosophila*. Acute dysfunction of STIM1 in the fat storage tissue triggers hyperphagia in flies [43]. These studies suggested that CRAC channels are critically contributed to the Ca^2+^ influx in adipogenesis and obesity.

## Transient receptor potential (TRP) channels in adipogenesis

Another major class of calcium-permeable channels is TRP family. TRP channels are normally non-selective to Ca^2+^ and sodium (Na^+^) permeation [44]. The structure of TRP channels is composed of six transmembrane (TM) domains, with both N-termini and C-termini located in the cytosol and a loop between TM5 and TM6 formed a pore for ion entry [45–49]. The TRP superfamily is further classified into TRPV (Vanilloid), TRPA (Ankyrin), TRPC (Canonical), TRPP (Polycystic), TRPM (Melastatin), TRPN (NomPC), and TRPML (Mucolipin), according to their primary amino acid sequences [50,51]. TRP channels are widely expressed and have a variety of physiological functions, such as detection of various mechanical and chemical stimuli in sensory transduction such as vision, hearing, olfaction, taste, touch, pain and thermosensation [52]. To date, accumulative evidence have shown that several TRP channels are involved in adipogenesis and function, suggesting that these TRP channels could be potential targets for human obesity treatment and prevention [53]. In the next session of the current review, we provided a brief introduction to the recent progress of TRP channels in adipogenesis and function.

## TRPV family in adipogenesis

In terms of TRPV family, several TRPV channels have been reported to be expressed in adipocytes and play important roles in proliferation, differentiation, thermogenesis of adipocytes and obesity.

In the past years, TRPV1 has been studied for its involvement in adipocyte differentiation and energy metabolism, which further related to obesity management. As the first identified TRP channels, TRPV1 is activated by capsaicin, a highly selective agonist of TRPV1, or when the surrounding temperature is higher than 43°C. TRPV1 is expressed in 3T3-L1 preadipocytes and adipose tissue both in animals and humans. TRPV1 has also been reported to play a key role in the regulation of food intake and glucose homoeostasis in WAT during the development of obesity [54]. Activation of TRPV1 by dietary capsaicin treatment induces a significant increase of Ca^2+^ influx and impaired differentiation in 3T3-L1 pre-adipocytes [55], which is probably through the calcineurin pathway [56]. In mature adipocytes, downregulation of TRPV1 significantly reduces the calcium increase which is activated by capsaicin during adipogenesis [55]. Lacking TRPV1 exacerbates obesity and promotes insulin resistance, which is associated with diabetes and ageing [54]. However, dietary capsaicin treatment has also been reported to prevent high fat diet (HFD)-induced obesity in wild-type (WT) mice *in vivo*, but not in TRPV1 knockout mice [55,57]. Recently, TRPV1 has been shown to play a key role in regulating the browning of WAT, which could be a novel strategy to counteract obesity [58]. It has been reported that capsaicin increases intracellular Ca^2+^ level of adipocytes and promotes the browning of WAT. Moreover, activation of TRPV1 increases the expression level of thermogenic genes, such as UCP1, and induces the browning process in 3T3-L1 pre-adipocytes [59]. In BAT, TRPV1 activation is involved in the stimulation of metabolism and energy expenditure to protect against obesity [60]. Similarly, monoacylglycerol, another TRPV1 agonist, increases UCP1 expression in BAT and significantly reduces the mass of visceral fat in HFD-treatment mice [61]. However, knockout of TRPV1 prevents HFD-treatment-induced obesity [62] and obesity-induced hypertension [63]. Furthermore, the lack of TRPV1 promotes obesity and induces leptin and insulin resistance, which in turn, resulted in increased food intake and decreased physical activity [54]. These contradictory data indicate the complicated effects of TRPV1 in regulating obesity. Therefore, TRPV1 could be a potential target for obesity management and drug application.

TRPV3 often form functional heteromeric channels with TRPV1 [64], which also shows similar effects in regulating adipogenesis and obesity with TRPV1 [65]. TRPV3 could be primarily activated by a high noxious threshold which is over 50°C and then becomes responsive to warm temperatures [66]. It has been reported that the expression level of TRPV3 was decreased in visceral adipose tissue in HFD‐treatment mice, ob/ob and db/db mice [67], and also reduced in subcutaneous WAT and interscapular BAT in HFD-treated and db/db mice, which is similar to TRPV1 [68]. HFD feeding increases TRPV3 expression in the medial nucleus tractus solitarius (mNTS) and hypoglossal nucleus (HN) in rats, which is accompanied by a reduced expression of proopiomelanocortin (POMC) and resulted in increased food intake and a gain of body‐weight [69]. It has been reported that activation of TRPV3 prevented adipogenesis in 3T3-L1 preadipocytes and played an anti-adipogenic role *in vivo* [65]. Activation of TRPV3 by its agonist, such as diphenylborinic anhydride and (‐)‐epicatechin, prevents adipogenesis in 3T3‐L1 pre-adipocytes. Besides, chronic activation of TRPV3 prevented adipogenesis and weight gain in mice. However, the detailed role of the TRPV3-mediated Ca^2+^ influx in adipogenesis has not been fully understood.

TRPV2 and TRPV4 function as an osmo- and/or mechano-sensor, which could be activated by hypotonic solution or mechanical stimulation [70–73]. TRPV2 has also been reported to be expressed in both WAT and BAT [68,74], which can be activated by noxious heat with the threshold above 52°C [75]. The expression level of TRPV2 is higher in mature adipocytes than in pre-adipocytes. Additionally, TRPV2 has been reported to play a role in adipocyte differentiation. It is reported that knockdown of TRPV2 reduces the differentiation of human white adipocytes [76]. TRPV2 has been proved to participate in thermogenesis and brown adipocyte differentiation [77,78]. The knockout of TRPV2 significantly decreases the mRNA expression levels of thermogenic genes, including PGC1α and UCP1. TRPV2 knockout mice have increased body weight, which is more fat upon HFD-treatment, accompanied by accumulated lipid droplets and enlarged sizes of brown adipocyte [77]. Moreover, activation of TRPV2 has been reported to prevent the brown adipocyte differentiation in mouse brown pre-adipocytes, which is probably via a calcineurin pathway [78]. These findings suggested that the TRPV2-mediated Ca^2+^ influx plays an important role in BAT differentiation and thermogenesis. And TRPV2 could be a target for preventing human obesity and other metabolic-related diseases [77–79]. However, the detailed mechanisms of TRPV2 in adipocyte differentiation are still unknown, which needs further studies in the future.

TRPV4 has been reported to be highly expressed in adipose tissue [80], such as WAT and BAT in mouse, as well as in human adipocytes, and the expression level of TRVP4 is higher in WAT than in BAT [81]. In adipocytes, TRPV4 functions as both hypotonic and major Ca^2+^ permeable channels. The amount of Ca^2+^ influx through one single TRPV4 channel is assessed to be around 100 times more than that of the L-type Ca^2+^ channel, which in turn to simulate various Ca^2+^-dependent signalling cascades [82]. Downregulation of TRPV4 did not affect adipogenesis in 3T3-F442A adipocytes. However, the administration of GSK205, an inhibitor of TRPV4, up-regulates the expression level of thermogenic genes such as *Ppargc1a* and *Ucp1*, which further promotes the browning process in 3T3-F442A adipocytes. Besides, pharmacological inhibition or deletion of TRPV4 also activates energy expenditure and protects mice from HFD-induced obesity *in vivo* [81]. These results suggested that inhibition of TRPV4 promotes browning of WAT by reducing the intracellular Ca^2+^ level [81]. However, knockout of TRPV4 has been proved to increase weight gain and promotes obesity during HFD-treatment in mice [83]. These results suggested a contradictory role of TRPV4 in adipogenesis and obesity. Therefore, further investigation is necessary to understand the role of TRPV4 in regulating Ca^2+^ influx, adipogenesis and obesity.

## Other TRP members in adipogenesis

Several TRPC (TRPC1, 4, 5) channels have been reported function as SOCs, by interacting with the key players of SOCE, such as ORAI1 and STIM1, which we have discussed previously. TRPC1 usually forms a tetrameric complex with TRPC4 or TRPC5 and interacts with each other to stimulate the intracellular Ca^2+^ signalling pathway. The homomeric TRPC1 alone does not functionally work on the plasma membrane [84]. It has been recently reported that increasing the level of extracellular adenosine triphosphate (ATP) induces Ca^2+^ influx in adipocytes via CRAC channels, such as ORAI1 and STIM1 [85]. The activation of ORAI1 not only induces the SOCE, but also stimulates the translocation of TRPC1 onto the plasma membrane by which mediates an additional Ca^2+^ influx [86]. TRPC1, TRPC4, TRPC5, and TRPC6 have been reported to be expressed in both preadipocytes and adipocytes, suggesting that these TRPCs may participate in adipogenesis [87]. Indeed, it has been proved that TRPC1 negatively regulates HFD-treatment induced obesity [88]. Besides, TRPC4 and TRPC6 were differentially expressed in pre‐adipocytes and mature adipocytes. These results suggested that TRPCs may play critical roles in adipogenesis [88]. Since TRPCs allow both the entry of Ca^2+^ and Na^+^ ions, the exact role of TRPCs mediate signalling in adipogenesis and obesity needs further studies.

Other TRP channels, such as TRPM8 and TRPPs, have also been reported to be involved in adipogenesis and obesity. TRPM8 is known as a cold sensing channel with a temperature threshold lower than 26-28°C [89], which can be activated by menthol or icilin [89,90]. TRPM8 has been reported to be functionally presented in BAT of the mouse, and the expression levels of TRPM8 are significantly increased during adipocyte differentiation [91]. Activation of TRPM8 in adipocytes by menthol up-regulates UCP1 expression and requires protein kinase A activation, which in turn enhances the BAT thermogenesis and browning of WAT in mice [92,93]. Besides, TRPM8 also expresses in a cell line of human white adipocyte. Activation of TRPM8 induces UCP1 expression, WAT browning, mitochondrial activation, and heat production [91]. TRPM8 has also been proved to be involved in the regulation of clock and clock-controlled genes in BAT [94].

TRPP is a type of non-selective ion channel, which has been proved to be associated with autosomal dominant polycystic kidney [95]. TRPP has three family members, TRPP2, TRPP3, and TRPP5. TRPP2, also known as PKD2 or polycystin‐2, has been reported to be expressed in adipose tissue, and the expression level of TRPP2 in mature adipocytes is higher than in pre-adipocytes [87,95]. However, the role of TRPP2 in adipogenesis has not been investigated yet. TRPP3 plays a role in BAT differentiation and thermogenesis [96]. Down-regulation of TRPP3 suppresses the expression of thermogenic genes, such as UCP1 and PGC1α, and attenuates the mitochondrial respiration but with no effect on adipogenesis. These results suggested that TRPP3 may facilitate BAT differentiation by enhancing mitochondrial function [96]. Taken together, it has been proved that some TRP channels are involved in adipogenesis and adiposity. However, the direct role of these channels in adipogenesis and obesity needs to be further investigated.

## Ca_v_ channels in adipogenesis

Ca_v_ channels, namely voltage-gated calcium channels, are one of the major calcium-permeable channels, which are widely expressed in numerous cell types such as neurons and adipocytes, and play important roles in regulating cellular processes, including release of neurotransmitters in neurons, and activation, differentiation and proliferation in adipocytes, respectively [97,98]. Ca_v_ channels are grouped into three subtypes: L-type high-voltage-activated (HVA) Ca_v_ channel is encoded by Ca_v_1; other HVA Ca_v_ channels, such as P/Q-type, R-type, and N-type, are encoded by Ca_v_2; and low-voltage-activated (LVA) T-type Ca_v_ channel is encoded by Ca_v_3. Some of these Ca_v_ channels have been proved to be involved in the regulation of intracellular calcium homoeostasis and adipocyte differentiation. The presence of L-type Ca_v_ has been reported to be expressed in adipocytes by experimental and clinical studies. L-type Ca_v_ is also involved in high extracellular Ca^2+^-stimulated adipogenesis in porcine BMSCs [34], because the effects of extracellular calcium on adipogenesis can be inhibited by an L-type Ca_v_ blocker, Nifedipine. Besides, the distribution of L-type Ca_v_ and the basal levels of intracellular calcium have been proved to be regulated by the growth hormone in rat adipocytes [99,100]. The T-type Ca_v_ has been firstly reported to be expressed in 3T3-F442A pre-adipocytes [101]. Then Ca_v_3.1 has been proved to be expressed in cultured pre-adipocytes and 3T3-L1 pre-adipocytes, and the protein-expression level of Ca_v_3.1 is significantly decreased in the differentiated adipocytes [102]. It has been confirmed that the selective block of T-type Ca_v_ by NNC55-0396, a T-type Ca_v_ inhibitor, inhibits the proliferation in pre-adipocytes [102]. It has been further confirmed that mice lacking Ca_v_3.1 or treated with TTA-A2, another selective blocker of T-type Ca_v_, are resistant to HFD induced increase of fat mass and body weight gain [102]. These results suggested that Ca_v_ channels, such as Ca_v_3.1, appear to be potential targets for the prevention and treatment of obesity.

## P2 receptors in adipogenesis

P2 receptors are one type of purinergic receptor, which is in response to the release of ATP. They have been classified into five subclasses, including ionotropic receptors such as P2X, P2Z, and metabotropic receptors such as P2Y, P2 U, and P2 T. P2 receptors have been reported to be involved in several cellular functions, such as vascular reactivity, cytokine secretion, and cell proliferation and migration [103]. However, the effects of purinergic stimulation seem to vary between cell types. A series of evidence have reported that extracellular ATP induces various cellular functions in both adipocytes and pre-adipocytes. In WATs, external ATP has been reported to be involved in glucose transport [104], glycogen synthase [105], lipolysis [106] and cytosolic Ca^2+^ homoeostasis [107]. In BATs, it has been reported to modulate membrane trafficking [108], cytosolic Ca^2+^ homoeostasis [109], and cell proliferation [110]. In 3T3-L1 pre-adipocytes, the extracellular ATP has been demonstrated to regulate hormone-induced adipocyte differentiation, which is mediated by P2Y receptors without affects pre-adipocyte proliferation [111]. It has been recently reported that knockdown of CD36, a membrane protein that has been demonstrated to participate in the progression of adipogenesis, resulted in a reduction of adipocyte differentiation and downregulation of purinergic receptor P2X7 expression, suggesting that the suppression of adipogenesis mediated by CD36 is probably via P2X7 pathway in 3T3-L1 cells [112]. Although P2 receptors have been proved to play a role in adipogenesis, the underlying mechanisms are still needed for further investigation.

## Concluding Remarks

**Figure 2.**
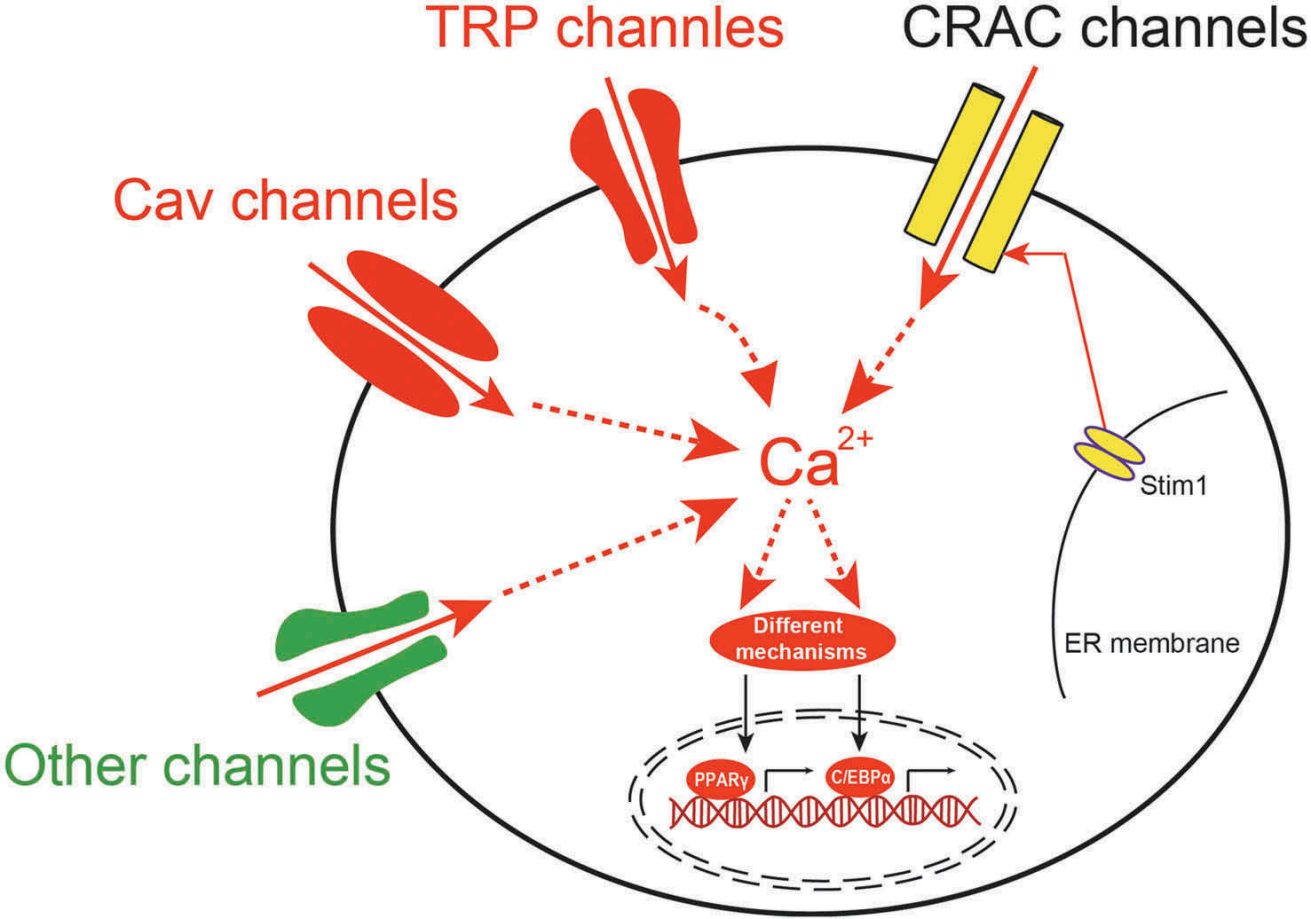
The schematic diagram of the involvement of Ca^2+^-permeable ion channels in adipogenesis

In the present review, we summarized the recent research progress of Ca^2+^-permeable channels, such as CRAC channels, TRPs, Ca_v_ and other channels in adipogenesis (Figure 2). Disturbance in Ca^2+^ homoeostasis through manipulating these calcium channels affects the downstream signalling pathways which in turn promotes or inhibits adipocyte differentiation and subsequently affects obesity. However, there are still some open questions that should be carefully considered in future studies. First, how do pre-adipocytes and mature adipocytes balance the Ca^2+^ fluxes among different components to maintain Ca^2+^ homoeostasis? Second, is there any other Ca^2+^ permeable channels involved in adipogenesis and obesity, such as high-voltage-activated Ca_v_ channels and NMDA receptors. Third, is the effect of cytosolic Ca^2+^ on adipocyte differentiation phase-dependent? Further in-depth studies are required to determine the best therapeutic targets of calcium channels for clinical treatment of obesity and related metabolic disorders. Such studies could pave the way for new clinical approaches to treating human obesity and related metabolic diseases.

In pre-adipocytes or bone marrow mesenchymal stem cells (BMSCs), several different types of Ca^2+^-permeable ion channels, such as Ca^2+^ release-activated Ca^2+^ (CRAC) channels, transient receptor potential (TRP) channels, voltage-gated calcium (Cav) channels and other channels, are expressed and contributed in adipogenesis. Upon these channels activation by respective stimuli, extracellular Ca^2+^ entry into the cytoplasm. And the increase of cytosolic Ca^2+^ modulates adipogenesis by promoting transcription via different mechanisms. PPARγ: peroxisome proliferator-activated receptor γ; C/EBPα: CCAAT-enhancer-binding protein α.
